# A review of natural products targeting tumor immune microenvironments for the treatment of lung cancer

**DOI:** 10.3389/fimmu.2024.1343316

**Published:** 2024-02-01

**Authors:** Pengyu Yao, Su Liang, Zhenying Liu, Cuiping Xu

**Affiliations:** ^1^ Department of Traditional Chinese Medicine, Jinan Maternity and Child Care Hospital Affiliated to Shandong First Medical University, Jinan, China; ^2^ Institute of Chinese Materia Medica, China Academy of Chinese Medical Sciences, Beijing, China; ^3^ Department of Nursing, The First Affiliated Hospital of Shandong First Medical University (Shandong Provincial Qianfoshan Hospital), Jinan, China

**Keywords:** natural compounds, lung cancer, tumor immune microenvironments, immune cells, immune cytokines, review

## Abstract

Lung cancer (LC) produces some of the most malignant tumors in the world, with high morbidity and mortality. Tumor immune microenvironment (TIME), a component of the tumor microenvironment (TME), are critical in tumor development, immune escape, and drug resistance. The TIME is composed of various immune cells, immune cytokines, etc, which are important biological characteristics and determinants of tumor progression and outcomes. In this paper, we reviewed the recently published literature and discussed the potential uses of natural products in regulating TIME. We observed that a total of 37 natural compounds have been reported to exert anti-cancer effects by targeting the TIME. In different classes of natural products, terpenoids are the most frequently mentioned compounds. TAMs are one of the most investigated immune cells about therapies with natural products in TIME, with 9 natural products acting through it. 17 natural products exhibit anti-cancer properties in LC by modulating PD-1 and PD-L1 protein activity. These natural products have been extensively evaluated in animal and cellular LC models, but their clinical trials in LC patients are lacking. Based on the current review, we have revealed that the mechanisms of LC can be treated with natural products through TIME intervention, resulting in a new perspective and potential therapeutic drugs.

## 1 Introduction

Cancer poses a significant clinical, social, and economic burden, and lung cancer(LC) is one of the leading culprits. LC is a highly prevalent disease and remains the major cause of global cancer-associated mortality. In 2020, LC is predicted that there were around 2.21 million newly diagnosed instances of cancer, accounting for approximately 11.4% of all reported cancer cases. Additionally, there were approximately 1.80 million fatalities attributed to cancer, representing approximately 18% of all reported cancer-related deaths (1). LC can be classified into two main histological categories: non-small-cell lung carcinoma (NSCLC) and small-cell lung carcinoma (SCLC). More than 80 to 85% of LC cases are NSCLC, which is more common than SCLC (2). Treating LC has always been a tricky and complex process. Managing LC includes surgery, chemotherapy, radiation therapy, biological therapies, immunotherapies, and targeted therapies. Since the turn of the century, the cancer treatment landscape has dramatically changed, leading to substantial improvements in outcomes for patients (3). Immunotherapy has shown promising efficacy in regenerating and repairing tumor-specific immune responses by targeting both the activation and recovery of immune cell function. Immunotherapy has thus brought new treatment options for cancer treatment, including LC. With the rapid advancement of tumor immunotherapy, LC treatment has gradually transitioned into a new era of immunotherapy.

In the process of transforming normal cells into tumor cells, the internal and external environment of tumor cells is closely related to the course of the disease. This highly structured and closely related complex ecosystem is called the tumor microenvironment (TME). TME includes both cancerous and non-cancerous cells. Non-cancerous cells (such as immune cells, endothelial cells, fat cells, etc.) and extracellular matrix in TME have extremely important effects on the biological behavior of cancerous cells. Since immune cells represent a large fraction of the TME, they play a key role in mediating pro- and antitumor immune responses (4). The tumor immune microenvironment (TIME) is often mentioned and studied independently. The TIME includes tumor cells, immune cells, immune cytokines, etc. The TME contains different types, in which TIME is like a battlefield between the tumor and immunity, mostly caused by the interaction of diverse tumor cells with immune cells. The characteristics of the TIME are intrinsically related to the efficacy of immunotherapy. Identifying targets that modulate immune metabolism helps to maximize the efficacy of cancer therapies (5). Immunotherapy alters the TIME and restores the ability of anti-tumor immune cells to kill tumors (6). Tumors can evade immune destruction by constructing an immunosuppressive microenvironment.

Natural products with novel structures, multi-targets, multi-pathways, few toxic and side effects, and diversified biological activities have numerous kinds of impacts on the treatment of diseases and are some of the most important sources of new drug research and development. Natural compounds and their derivatives show strong anti-tumor activity in many types of cancer, and their effects are being confirmed through various experimental studies, including on lung, breast, colon, and prostate cancer (7). Natural products have a significant impact on cancer treatment by modulating TME and different signaling pathways (8). The investigation of novel natural-product-derived compounds with the potential to modulate tumor immunotherapy has emerged as a prominent and globally recognized area of research (9). Many natural products have immune-modulating properties that could revolutionize the LC landscape. This work provides a comprehensive review of the processes by which natural compounds are regulated during the TIME of LC. Natural compounds will undoubtedly be essential to the control of TIME in LC treatment. This investigation provides a theoretical basis for natural compounds that regulate the TIME in LC treatments and presents innovative research ideas for exploring and developing new anticancer drugs.

## 2 Overview of tumor immune microenvironment and lung cancer

The interaction and co-evolution between tumor cells and their microenvironments causes LC to occur and develop. The crucial involvement of the TME in the onset and advancement of LC has been widely recognized during the past decade (10). The TIME is the “battlefield” where tumour cells and immune cells interact. Certain immune cells can detect and eliminate tumor cells, whereas tumor cells can also influence their microenvironment by producing chemicals that signal other cells and cause immunological tolerance. It is well established that neighboring immune cells and interactions across cell types influence the biological phenotypes and behaviors of malignant T cells. This complex tumor ecosystem is collectively known as the TIME (11). The immune system and tumor cells have complex interactions throughout the initiation and development of cancer (12). Different immune cells possess distinct surface markers, secretory factors, and corresponding immune functions. Immune cells are the primary players in TIME, affecting and controlling the occurrence and development of tumors in a complex and precise manner. Based on the various immune cell types identified in the TIME, this study examines the function of LC-associated immune cell types.

### 2.1 Helper T cells

Helper T (Th) cells are the largest subpopulation of T cells and important components of the TIME. The cluster of differentiation (CD) is a nomenclature system that identifies and classifies cell surface molecules present in leukocytes. CD is a common cellular marker for immune antigen recognition, with CD4 (markers for Th cells) and CD8 (markers for cytotoxic T cells) being the most common. Th cells and regulatory T cells (Tregs) are commonly known as CD4 T cells due to the presence of CD4 molecules on their cell surfaces. The activation of quiescent naive CD4^+^ T cells (Th0) is assisted by their identification of antigen-major histocompatibility complex molecules and appropriate co-stimulation (13). Naive CD4^+^ T cells differentiate into one of several Th cell subsets, depending on specific antigens and cytokines in the immune microenvironment. Th cells play a pivotal role in regulating adaptive immune responses at epithelial sites by the secretion of a diverse range of cytokines, which serve to attract and modulate the function of various other immune cells (14). Different subtypes of Th cells produce different cytokines and perform different immune functions. In 1986, Mosmann discovered the mysterious veil of the Th1 and Th2 subsets (15), and since then, other cell subpopulations have also been discovered, such as Th3, Th9, Th17, Th22, Tfh, etc. In the TIME of LC, Th1, Th2, Th9, and Th17 tend to be of higher concern.

Th1 cells are primarily responsible for regulating cellular immune responses; secrete immune cytokines; and promote the activation and proliferation of macrophages, cytotoxic T cells (CTLs), and natural killer (NK) cells. Th1 responses are overrepresented in LC (16). When tumor necrosis factor (TNF)-α and interferon (IFN)-γ levels are high, IL-2 levels are low, and these are Th1-biased cytokines in LC. The higher expression of TNF-α is associated with a low risk of LC progression (17). IFN-γ can cause tumor cells to evade immune surveillance in LC (18). IL-2 is considered an effective treatment option for activating the anti-tumor immune response. The validation of the therapeutic efficacy of increasing the activity of NK cells and T cells through IL-2 signaling has been confirmed as a novel approach for SCLC (19). In fact, the role of Th1 in lung cancer is mainly summarized in three aspects: the secretion of regulatory cytokines, the regulation of the immune response of other immune cells and the homeostasis of Th1/Th2 cells.

Th2 cells are associated with pro-tumor activity, mediating this function by producing various cytokines. IL-6, IL-10, and IL-4 are considered the main Th2 immune cytokines, mainly encouraging tumor growth by hindering the host’s immune system. IL-4 influences tumor-associated macrophage polarization in LC. Signal Transducer and Activator of Transcription 6 (STAT6) deficit leads to a decrease in IL-4 levels, therefore resulting in a diminished differentiation of macrophages into M2 macrophages (20). An increase in IL-6 levels can lead to molecular targeted drug resistance in LC, and these levels might be useful as a prognostic marker in patients with NSCLC (21). LC with mutations in the epidermal growth factor receptor (EGFR) exhibits a deficiency in IL-10, resulting in the inadequate induction of CD39 expression in CD8+ T cells, commonly referred to as cytotoxic CD8+ T lymphocytes or CTLs. Furthermore, IL-10 plays a crucial role in enhancing CD8+ T-cell-mediated cytotoxic functions, which depends on the presence of CD39 (22). Th1 cells are involved in the promotion of the anti-tumor immune response, while Th2 cells counteract the activity of Th1 cells. Tumorigenesis and relapse may depend on alterations to Th1/Th2 cytokine homeostasis. Accordingly, in patients with LC, it can be found that Th2 cytokine levels increase, whereas Th1 levels decrease (23).

Th9 cells are characterized by the secretion of IL-10 and IL-9. Th9 cells mainly secrete cytokine IL-9 to act on other cells to play anti-tumor immunity. It has been reported to be effective in eliminating solid tumors, and exhibit remarkable anti-tumor properties compared with Th1 and Th17 cells (24). In addition, it has been reported that Th9 cells induce EMT in LC, thereby promoting migration and metastasis (25). According to the reports mentioned above, Th9 cells have a dual role in tumorigenesis, as they can play both anti-tumor and pro-tumor functions. Therefore, the inducing effect of drugs on Th9 cell function is a promising research direction.

Th17 cells and related immune cytokines can promote either tumorigenesis or tumor suppression, but the relevant mechanism is still unknown. The interaction between Th17 cells and Tregs is crucial in regulating autoimmunity and cancer (26). It is abundantly clear that the balance between Th17 cells and Tregs is significantly disturbed in NSCLC (27). In fact, the intricate relationships between immune cells often play an unexpected role in TIME, but they are often mysterious and waiting to be revealed.

### 2.2 Cytotoxic T lymphocytes

It is generally recognized that CTLs, as the primary effector cells of cellular immunity in the human body, can detect and eliminate malignancies, making them an essential part of the adaptive immune system. The number of CTLs within the TME is a pivotal prognostic marker of cancer, and high-CTL tumors are more likely to respond to immunotherapy than low-CTL tumors (28). CD3 is a common surface marker of T cells, marking all T cells. CD8+ is used as a specific marker in CTLs, so it is often called the CD8+ T-cell. CD8+ T cells constitute a highly heterogeneous cell population among patients with LC (29). The mechanism by which CTLs kill tumor cells involves the following three aspects (1): the granular exocytosis pathway, via the synaptic exocytosis of cytotoxic granules that contain perforin and granzymes into the target, results in tumour cell destruction (2); the cytotoxic cytokine pathway, secretes cytokines, including IFN-γ and TNF-α (3); and CTLs are capable of eliminating target T cells via a chain reaction that results in apoptosis via the FAS ligand (FASLG) molecule (30, 31). Tumor heterogeneity determines that even the same immune cells play different roles at different TIME. The significance of CTLs in SCLC has been explored more thoroughly than in NSCLC. Insufficient anti-tumor immune responses are caused by a lack of pre-existing tumor-infiltrating T lymphocytes (TITLs), particularly CTLs, in the SCLC TME (32). In addition, SCLC cells have an immune-provoking impact on cytotoxic T lymphocytes, which upregulate the co-inhibitory receptors of CTLs, thus inducing T-cell exhaustion upon prolonged activation (33). As multicellular organisms, cell-cell interactions (CCIs) play an important role in maintaining homeostasis and coordinating physiological functions. CCIs between immunizations plays an irreplaceable role in TIME, although it is complex and mysterious.

### 2.3 Regulatory T cells

Tregs are a special subgroup of CD4+ T cells with immunosuppressive, which represent 5−10% of peripheral CD4+ T cells (34). Tregs represent an important immunosuppressive cell type in TIME, which inhibit the activation of other immune cells and maintain the immune system’s homeostasis. Tregs can inhibit anti-tumor immunity through two stages, hindering immunosurveillance against cancer development, and hampering effective anti-tumor immune responses in hosts (35). Tregs are the “culprit” that helps tumor cells evade the body’s immune surveillance. Tumor cells can use Tregs to achieve immune escape. In addition, Tregs are therapeutic targets and biomarkers that can predict the LC’s survival length and recurrence, particularly in circulation or regional lymph nodes (36). In NSCLC, Tregs are associated with tumor staging, therapeutic efficacy, and prognosis, infiltrating tissues. Tregs attenuate immunologic anticancer effects in NSCLC patients (37). Their inhibition mechanisms include the following: suppressive cytokines; IL-2 consumption; the antigen-presenting cell (APC) mediated pathway via cytotoxic T-lymphocyte-associated protein 4 (CTLA-4); the T-cell immunoreceptor with immunoglobulin and the ITIM domain (TIGIT); the metabolite-related mechanism via CD39/CD73; lymphocyte activation gene 3 (LAG3); contact-dependent suppression via programmed death ligand 1 (PD-L1); and other mechanisms (38).

### 2.4 Natural killer cells

NK cells belong to large granular lymphocytes and are the third largest lymphocyte population after T cells and B cells. They serve as the primary effector cells regarding innate immunity and are highly heterogeneous in the TIME. The main role of NK cells can be summarized in two aspects: One acts as an important part of tumor immune surveillance. Many receptors on NK cells tightly regulate their activation and allow them to distinguish between ‘normal’ and ‘dangerous’ cells (39). The second is directly lysing tumor cells in a similar manner to activated CTLs. NK cells possess two primary cytotoxic mechanisms: granulocyte apoptosis mediated by perforin and granzyme and antibody-dependent cell-mediated cytotoxicity (ADCC) (40). Furthermore, as cytotoxic innate lymphoid cells, NK cells generate inflammatory cytokines and chemokines, including TNF-α and IFN-γ (41). The involvement of NK cells in LC is not fixed but rather exhibits alterations throughout the progression of the disease. The anti-tumor effect of NK cells varies according to the stage of LC. In the early stages of tumor development, NK cells with high cytotoxicity and survival can efficiently eliminate tumor cells. The NK cells involved in tumor promotion enter a mildly dysfunctional condition and reach a state of equilibrium with the tumor cells. Tumor cells resist NK cell-mediated immunosurveillance and attempt to escape from them as the tumor develops (42). The interaction between NK cells and LC is reciprocal. The colony-forming level and cytotoxicity of NK are significantly negatively correlated with LC, and the colony-formation and cytotoxicity of NK cells in LC patients were significantly lower than those in healthy people (43). There is increasing evidence suggesting that improving NK cell functioning may induce tumor regression, and immunotherapy targeting NK cells may be an effective strategy in LC treatment (44, 45).

### 2.5 Tumor-associated macrophages

Tumor-associated macrophages (TAMs) are significant constituents of the TIME, exhibiting a wide range of supporting and inhibitory influences on the growth, advancement, and metastasis of LC (46). In LC, TAMs are involved in participating in the growth, angiogenesis, metastasis, and invasion of cancer cells. Thus, TAMs are regarded as a potentially effective therapeutic target for LC (47). According to a recent study, in LC and LC-related disorders, cytokines and chemokines that are released through interactions between TAMs and tumor cells substantially stimulate antiapoptotic, hyperproliferative, and metastatic responses (48). TAMs can be divided into three subsets: the classical subtype M0 (non-polarized or neutral), M1 (anti-tumor), and M2 (pro-tumor) macrophage. TAMs have the opposite functional potential, which may depend on the cytokine environment in the TIME. The M1- and M2-like gene signature expressions are not mutually exclusive in early LC, as both gene expression profiles can be displayed simultaneously by the TAMs (49). However, TAMs in early-stage lung adenocarcinoma appear to express genes promoting tumorigenesis, and they are not known to have distinct M1 and M2 macrophage polarization (50). The value of TAMs in LC TIME is certain, but their specific mechanism remains to be fully explored and studied.

### 2.6 Cancer-associated fibroblasts

Over the past decade, the idea that Cancer-associated fibroblasts (CAFs) are immunosuppressive cells has been widely accepted. CAFs, as a most prominent and abundant cell population, are a major component of the TME and account for nearly 70% of cells in tumor tissues (51). CAFs perform a wide range of functions, such as the secretion of inflammatory ligands, growth factors, and extracellular matrix (ECM) proteins that enhance drug resistance, immune antagonistic effects, and tumor development (52). In addition, CAFs are actively involved in cancer progression, in the way that through complex interactions with other cell types in the TIME (53). As prospective therapeutic targets, T-cell exclusion in NSCLC is governed by the distinct ECM profiles and spatial distribution of CAFs (54). Furthermore, CAFs serve as a prognostic indicator for immunotherapy efficacy in non-small cell lung cancer (55). The ‘Reverse Warburg Effect’ in LC is supported by research, but it is limited to certain tumor cell lines and can be modified by different CAFs (56). The complex role and differential efficacy of CAFs in the TIME are certainly fully reflected in LC, but this needs to be further explored.

### 2.7 Tumor-infiltrating B cells

Although T cells have been the primary focus of tumor immune response studies in recent years, B cells also play an important role in tumor immunity. B cells, which continuously comprise a significant cellular component of the TME, might play an essential function in tumor immunity (57). B-cell density is considered a new prognostic biomarker of NSCLC patient survival (58). LC progression is significantly regulated by tumor-infiltrating B (TIL-B) cells. However, the role of TIL-B cells in human cancer, that is, whether they are anti-tumor or pro-tumor, depends on the tumor type. In patients with NSCLC, there is evidence that TIL-B cells have an anti-tumor effect. Anti-tumor immunity is mediated through the maintenance of structure and function of tertiary lymphoid structures (TLSs), the production of tumor-specific antibodies by TIL-B cells, and the promotion of T-cell responses; all of these mechanisms are associated with favorable outcomes in LC (59). Given their ability to efficiently deliver antigens to CD4+ TILs and modify the CD4+ TIL phenotype, TIL-B cells represent a promising therapeutic target in SCLC (60).

### 2.8 Dendritic cells

Dendritic cells (DCs) are a heterogeneous leukocyte population comprising distinct subsets, and their strong ability to initiate and regulate adaptive immune responses is the basis for successful anti-tumor immune responses (61). Tumor-specific immune responses are initiated, programmed, and regulated by DCs. DCs possess a distinctive ability to initiate immunological responses by capturing antigens and subsequently processing them into peptides. These peptides are then presented to naive T cells in lymphoid tissues via major histocompatibility complex (MHC) molecules (62). DCs play a critical role in protecting against LC, and clinical trials have shown that their function decreases in LC patients (63). According to research, patients diagnosed with advanced NSCLC have not responded well to vaccination with DCs or DC/cytokine-induced killer (CIK) cells (64). Modulating DCs at the early stage of LC seems to be a feasible immunoanticancer strategy, as suggested by the above studies.

### 2.9 Mast cells

Mast cells (MCs) are unique tissue-resident immune cells of the myeloid lineage, that secretes a variety of cytokines involved in immune regulation. MCs are recognized as critically shaping tumor cell and TME behavior (65). MCs play a complex role in the TME by modulating different tumor biological events, such as cell proliferation and survival, invasiveness, angiogenesis, and metastasis. Furthermore, tumor-associated MCs can influence TME through their interactions with other tumor-infiltrating cells (66), such as Th cells. The value of MCs in different types of LC continues to be studied. Some studies suggest that a constant decrease in MCs may be implicated in the whole invasive process of lung adenocarcinoma (LUAD) (67). Moreover, research confirms that high MC abundance correlates with prolonged survival in early-stage LC patients (68). The expression of the four genes linked to resting MCs (RMCRGs) and resting MCs infiltration in NSCLC are positively correlated; the greater the risk score, the lower the expression of immune checkpoint inhibitor (ICI) and resting MCs infiltration (69).

### 2.10 Myeloid-derived suppressor cells

Myeloid-derived suppressor cells (MDSCs) are a diverse population of pathologically activated cells that have strong immunosuppressive properties (70). In the context of cancer, MDSCs are abnormally produced and recruited into the TME to aid in establishing an immunosuppressive microenvironment that facilitates tumor immune escape (71). MDSCs are closely connected to the prognosis of patients with LC (72). There is increasing evidence that MDSCs are involved in the development of LC and may be used to predict the efficacy of immune checkpoint blockade (ICB) treatments (73). MDSCs can be further studied as immunosuppressive regulators and therapeutic targets in LC.

The critical role of TIME in promoting tumor growth and metastasis is strongly established. The TIME has been utilized to analyze particular interactions between tumor cells and immune cells of various types, and it was found that changes in the TIME, represented by immune cells, affect the progression and metastasis of LC. Although the anticancer and pro-cancer effects of some immune cells are still controversial, the role of TIME in LC is well established. This also makes TIME a potential target for improved immunotherapy. Overall, TIME is a novel field facing major challenges and deserves further exploration.

## 3 Anticancer effects of natural products when targeting tumor immune microenvironments

**Figure 1:**
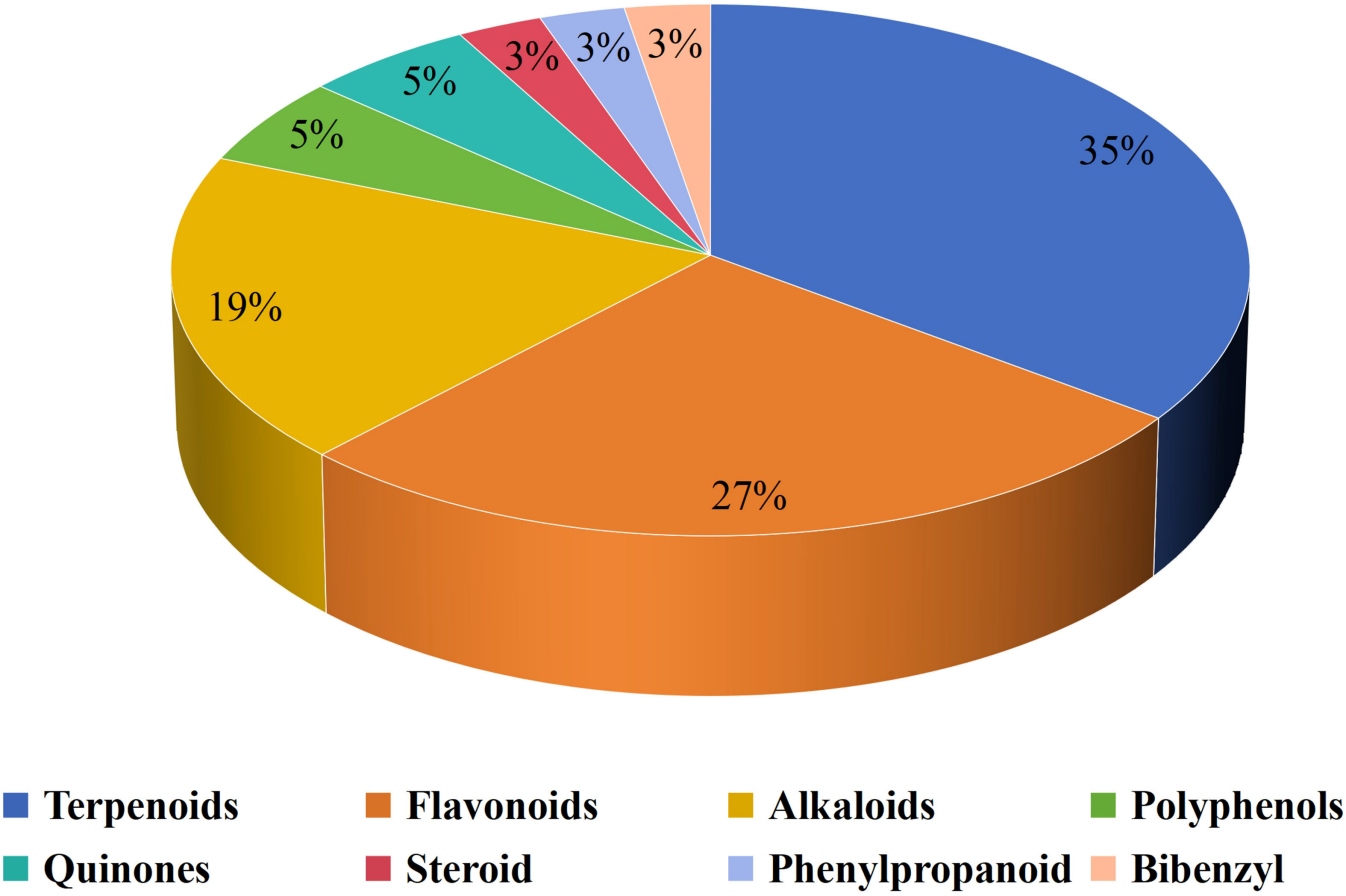
Distribution of different sub-classes of natural products.

Natural compounds play an important role in preventing and treating various cancers and have great research value and development prospects. The therapeutic role of natural compounds in cancer is complex and varied, and discoveries are constantly being updated. A wide range of natural compounds have been found to possess anticancer properties, demonstrating various actions such as inhibiting cell proliferation, promoting apoptosis, preventing metastasis and angiogenesis, regulating autophagy, reversing multidrug resistance, modulating immune responses, and enhancing the efficacy of chemotherapy both *in vitro* and *in vivo*. Natural compounds have an impact on tumor cells as well as TIME. Natural compounds are crucial to LC immunotherapy (74, 75). A total of 37 natural compounds have been reported to exert anti-cancer effects by targeting the TIME, **Figure 1** presents them.

### 3.1 Terpenoids

**Figure 2:**
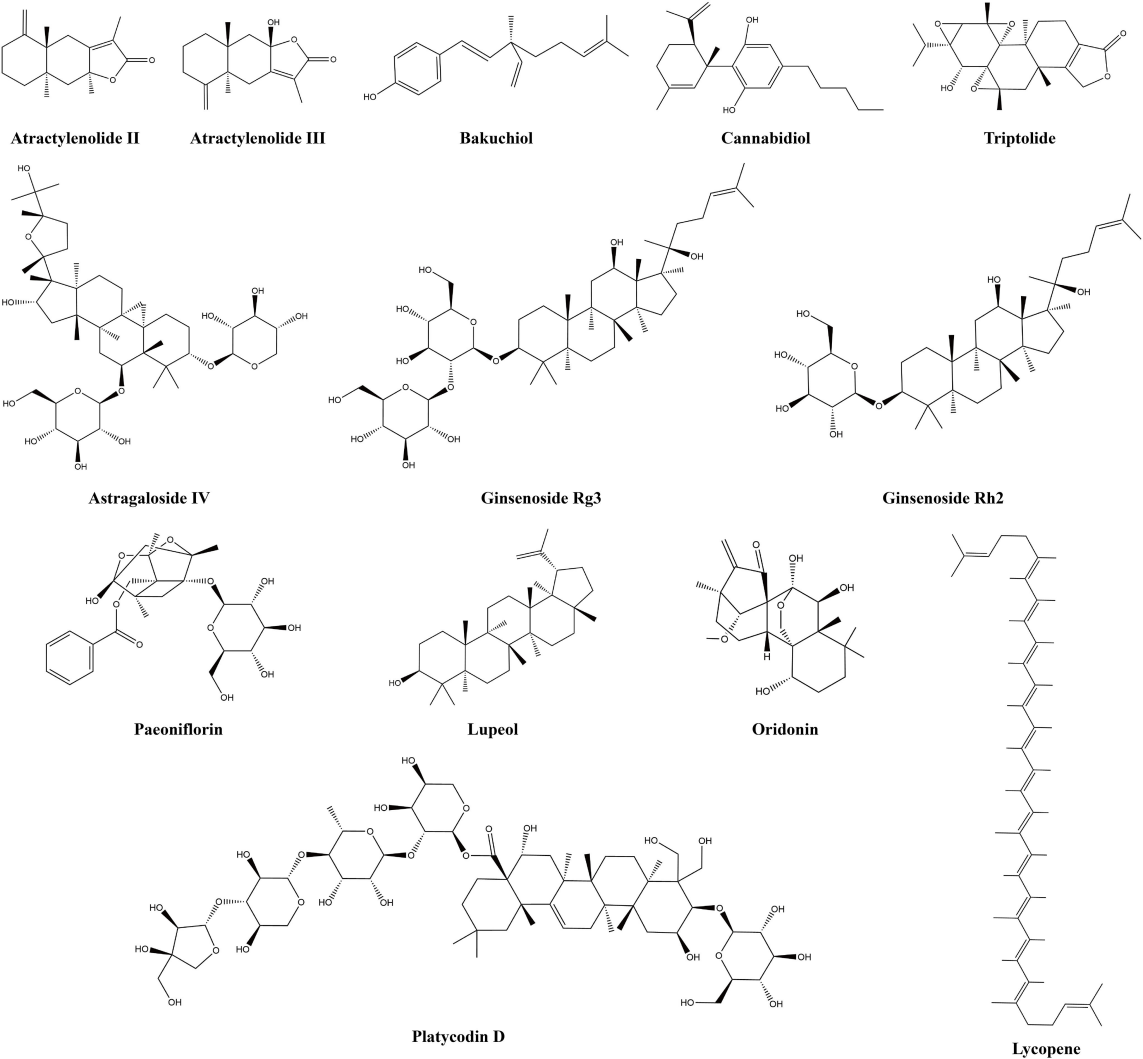
Chemical structures of terpenoids.

Terpenoids are a class of biologically active natural compounds that exhibit a wide range of pharmacological properties, rendering them a valuable reservoir of potential compounds for drug discovery (76). Recent efforts to study and produce terpenoids for their anticancer properties have shown promise and could lead to new possibilities for treating cancer. Many terpenoids are effective anti-LC agents that can delay the invasion and metastasis of lung carcinoma cells (77). Tumor-infiltrating immune cells and associated cytokines are crucial in facilitating or impeding tumor growth, a factor that has become an important focus in the treatment of LC due to the complexity of its development. This study found that 13 terpenoids exert their anti-LC effects by modulating the TIME. **Table 1** provides the basic information and mechanisms of these 13 terpenoids derived from recent studies on LC, while **Figure 2** presents their chemical structures.

**Table 1:** Basic information and mechanisms of 13 terpenoids from recent studies.

Number	Compounds	Subclass	Molecular Formula	Weight (g/mol)	Main Source	Experimental Model	Dosage (mg/kg; μm/L)	Dosing Cycle	Immune Cells/Immune Regulatory Cytokines and Protein	Effects	Reference
1	Astragaloside-IV	Terpenoids	C41H68O14	785	*Astragalus membranaceus* Bunge	A549 and H1299 cells	40; 40 and 80	21 d; 48 h	TAMs	Blocks the M2 polarization of TAMs	(78)
						Lewis lung carcinoma (LLC) mouse model; LLCs	40; 80 and 160 μg/mL	12 d; 24, 48, and 72 h	Tregs and CTLs	Decreases the number of Tregs, and increases the number of CTLS	(79)
2	Ginsenoside Rg3	Terpenoids	C42H72O13	785	*Panax ginseng* C. A. Mey.	LLCs	3, 10, 20, 50, 100, 200, 300, and 400 μg/mL	48 h	DCs; TNF-α, TGF-β and IFN-γ	Increases the number of DCs, induces the secretion of TNF-α or TGF-β, and promotes the production of IFN-γ by tumor cells	(80)
						A549 cells	5, 10, 20, 40, 80, and 160 μg/mL	12, 24 and 48 h	PD-L1	Downregulation of PD-L1 expression	(81)
3	Ginsenoside Rh2	Terpenoids	C36H62O8	622.9	*Panax ginseng* C. A. Mey.	A549 cells	0, 10, 20, 30, 40, 50, and 60	48 h	TNF-α	Upregulation of TNF-α expression	(82)
4	Atractylenolide II	Terpenoids	C15H20O2	232.32	*Atractylodes Rhizoma rhizomes*	A549 xenograft mouse lung cancer model; A549 cells	50; 2.5 and 5	21 d; 24 and 48 h	TAMs	Inhibits the M2 polarization of TAMs	(83)
5	Atractylenolide III	Terpenoids	C15H20O3	248.32	*Atractylodes Rhizoma rhizomes*	LLCs, H1703, H520, PC-9, A549, and H1299 cells	2, 4, 8, 16, 32, 64, 128, 256, and 512	24 h	IDO	Inhibits IDO expression	(84)
6	Cannabidiol	Terpenoids	C21H30O2	314.5	*Cannabis sativa* L.	A549 and H460 cells	3	48 h	ICAM-1	Upregulation of ICAM-1 expression	(85)
7	Triptolide	Terpenoids	C20H24O6	360.4	Tripterygium wilfordii Hook. F.	PC-9 and A549 cells	500 nm/L	24 and 48 h	IL-6	Inhibits the activation of the IL-6/STAT3 axis	(86)
8	Oridonin	Terpenoids	C20H28O6	364.4	*Rabdosia* rubescens	A549 xenograft mouse lung cancer model; A549 cells	10; 1, 3, and 10	4 weeks; 24 h	NK cells	Enhances NK cell activity	(87)
9	Lycopene	Terpenoids	C40H56	536.9	Tomato	LLC mouse model; LLCs	40; 40	12 d; 24 h	Th cells and CTLs; PD-L1, IL-1 and IFN-γ	Increases the CD4+/CD8+ cell ratio, upregulates IFNγ-expression, upregulates the levels of IL-1 and IFN- γ, reduces the levels of IL-4 and IL-10	(88)
10	Lupeol	Terpenoids	C30H50O	426.7	olives, mango, elm, Aloe vera (L.) Burm. f., Lupinus micranthus Guss.	H1299 cells	20	24 h	TAMs	Inhibits the M2 polarization of TAMs	(89)
11	Paeoniflorin	Terpenoids	C23H28O11	480.5	*Paeonia lactiflora* Pall.	LLC mouse model; LLCs	10, 20, and 40; 1, 3, 10, 30, and 100	21 d; 0, 12, 24, and 48 h	TAMs	Inhibits the M2 polarization of TAMs	(90)
12	Bakuchiol	Terpenoids	C18H24O	256.4	*Cullen corylifolium* (L.) Medik.	LLC mouse model	5, 15, and 40	15 d	TAMs; IL-1β, IL-2, IFN-γ, TNF-α, IL-4, IL-10 and PD-L1	Increases the number of cytotoxic immune cells (CD8+ T cells and M1 macrophages); decreases the number of pro-tumor immune cells (CD3+ T cells, Tregs, and M2 macrophages); upregulates IL-1β, IL-2, IFN-γ, TNF-α, IL-4, and IL-10 expression; downregulates PD-L1 expression	(91)
13	Platycodin D	Terpenoids	C57H92O28	1225.3	*Platycodon grandiflorus* (Jacq.) A. DC.	H1975 and H358 cells	10	24 h	PD-L1, IL-2	Decreases the level of PD-L1; triggers the extracellular release of PD-L1 and enhances IL-2 secretion	(92)

#### 3.1.1 Astragaloside-IV

Astragaloside-IV(AS-IV) is a cycloartane triterpene glycoside chemical and one of the bioactive ingredients isolated from *Astragalus membranaceus* Bunge. *Astragalus membranaceus* Bunge, also known as “HuangQi”, is widely used to treat various cancers in Traditional Chinese medicine (TCM). AS-IV, a well-investigated and significant natural compound in TCM, has exhibited its anti-tumor properties by impeding the growth, invasion, and metastasis of tumor cells in multiple cancer types (93). TAMs are key regulators of the complex interplay between cancer and the TIME (94) and a significant therapeutic target in LC. The M2 macrophage subpopulation is a cancer-promoting immune cell in TAMs, and M2 macrophage polarization invasions of tumor sites are frequently associated with tumor development and poor prognosis. By partially inhibiting the AMPK signaling pathway-mediated polarization of TAM M2 macrophages, AS-IV decreases the invasion, angiogenesis, and migration of tumor cells in LC (78). Tryptophan (Trp) is primarily catabolized by the enzyme indoleamine 2,3-dioxygenase (IDO) (95). IDO can degrade Trp, an important amino acid, into kynurenine and other downstream metabolites, which can decrease effector T-cell function and promote Treg differentiation. AS-IV exhibits a potent anticancer effect *in vivo* and can increase the immune response by lowering Tregs and raising CTLs activity, which may be linked to its ability to suppress IDO expression (79). Astragaloside demonstrates potential as a TIME intervention agent in LC, capable of enhancing the immune response and inhibiting tumor immune evasion through the regulation of various immune cells(include TAMs, CTLs and Tregs mentioned above).

#### 3.1.2 Ginsenosides (Rg3 and Rh2)

Ginsenosides are effective components of terpenoids extracted from *Panax ginseng* C. A. Mey. (it is also known as *Renshen* in TCM) that can inhibit tumors and enhance body immune functions. Ginsenosides are a class of tetracyclic saponins that can be categorized into two groups, namely dammarane and oleanane, based on the structure of their aglycones. Within the dammarane group, ginsenosides can be further classified into four types: protopanaxatriol (PPT), protopanaxadiol (PPD), oleanane (OA), and ocotillol (OCT). Ginsenoside Rg3 and ginsenoside Rh2 are both the PPD type. A large number of ginsenosides are effective in LC intervention, but only Ginsenosides Rg3 and Rh2 have been reported on regarding LC immunotherapy.

Although ginsenoside Rg3 and ginsenoside Rh2 compounds are similar in structure, the structure of ginsenoside Rg3 is more complex and well-studied. A meta-analysis of 12 studies confirmed that ginsenoside Rg3 has some efficacy advantages in improving immune function in NSCLC patients compared with other saponins (96). A monomer preparation of ginsenoside Rg3 was approved by the National Medical Products Administration in Chinese and is frequently used for various cancers, particularly in NSCLC, which can improve the survival rate and objective response rate in combination with chemotherapy in NSCLC patients (80).

Tumor cells can become immunogenic by changing from non-immunogenic to immunogenic in response to external stimuli, which is known as immunogenic cell death (ICD). Ginsenoside Rg3 can induce ICD in lung carcinoma cells via the mediated induction of apoptosis and a subsequent increase in the expression of the chaperone protein calreticulin (CRT) and heat shock proteins (HSPs) on the surface of lung carcinoma cells. In addition, Rg3 induces the secretion of TNF-α or TGF-β and promotes IFN-γ production by tumor cells (81). Weakening the tumor’s chemotherapy resistance through immune regulation is also an important part of anticancer research into natural products. PD-L1 is overexpressed in lung carcinoma cells/cisplatin-resistance (CR) cells compared with lung carcinoma cells, and ginsenoside Rg3 can suppress the PD-L1 expression and resume T-cell functions (82). The degree and success rate of anti-tumor immune response are determined by the synergistic effect of multiple cytokines in TIME, and TNF-α, TGF-β and IFN-γ are the cytokines that are often concerned. TNF-α is a central cytokine that contributes to malignant tumor progression in TIME. TNF-α upregulation has also been observed in ginsenoside Rh2-treated A549 tumour cells (97). Ginsenosides have garnered greater attention in the context of their regulatory effects on immune cytokines, in comparison to other natural products.

#### 3.1.3 Atractylenolides (Atractylenolides II and III)

Atractylenolides are sesquiterpenoids produced from the rhizomes of the plant *Atractylodes Rhizoma* (it is also known as *Baizhu* in TCM). These compounds have anti-tumor activity both *in vitro* and *in vivo*, making them an attractive option for treating a variety of cancers (98). Atractylenolides exhibit a diverse array of pharmacological properties, and their role in the treatment of LC has been proven (99). Atractylenolides mainly include Atractylenolide I, II, and III and Atracylon. Their pharmacological action is somewhat similar, possibly because of their shared tricyclic structure. In atractylenolides, atractylenolides II and III have been reported to be able to exert anti-cancer effects by intervening in TIME.

The promotion of tumor proliferation, angiogenesis, and metastasis is greatly influenced by M2 macrophage. By efficiently preventing M2 macrophage polarization, atractylenolide II prevents tumor cell metastasis *in vivo* as well as *in vitro*. The efficient inhibition of M2 macrophage polarization by Atractylenolide II mostly occurs through the activation of the STAT6 signaling pathway by inhibiting IL-4/IL-13 (83).

IDO has been recognized as a crucial protein checkpoint involved in the modulation of the TIME, hence facilitating tumor progression. By directly binding to the Jak3 protein, Atractylenolide III has demonstrated remarkable efficacy in blocking IFN-γ driven Jak3/STAT3 signaling pathway-dependent IDO activation (84).

#### 3.1.4 Cannabinoids

Cannabinoids are terpenophenolic compounds derived from the *Cannabis sativa* L. plant. Cannabidiol is a non-psychoactive cannabinoid, and clinical studies have reported that cannabidiol may cause surprising reactions in LC patients (100).

The expression of Intercellular Adhesion Molecule-1 (ICAM-1), a transmembrane glycoprotein belonging to the immunoglobulin superfamily (IgSF), is observed in tumor cells. ICAM-1 can impact tumor development by promoting adhesive between tumor and immune cells (101). Cannabidiol can increase lung tumour cell lysis caused by lymphokine-activated killer cells by upregulating ICAM-1 (85). Cannabinoid receptors are key targets for cannabinoid action and are expressed in tumour cells and TIME cells. The expression of the cannabinoid receptor has an impact on the development of cancer in different types of tumors. TME-derived Cannabinoid2 s NSCLC model (102). This provides a new path to discovering LC treatments using cannabinoids to intervene in the TIME.

#### 3.1.5 Triptolide

Triptolide is a diterpenoid of the abietane class that was extracted from *Tripterygium wilfordii* Hook.(also known as *Leigongteng* in TCM) Triptolide is the key ingredient of *Tripterygium wilfordii* Hook against cancers, it demonstrates substantial anticancer properties. The IL-6/STAT3 signaling pathway acts to drive the malignant progression of tumors, while strongly suppressing the antitumour immune response. Thus, target IL-6/STAT3 signaling pathway are poised to provide therapeutic benefit by stimulating antitumour immunity. The IL-6/STAT3 signaling pathway may play an essential role in the TIME; IL-6-mediated STAT3 activation in the TME inhibits the functional maturation of DCs, thus activating effector T cells and blocking the emergence of anti-tumor immunity in cancers (103). Triptolide can exert anti-tumor effects on lung carcinoma cells of NSCLC by inhibiting the activation of the IL-6/STAT3 axis (86).

#### 3.1.6 Oridonin

Oridonin is a biologically active diterpenoid molecule derived from the plant species *Rabdosia rubescens*. Multiple studies have provided evidence supporting the inhibitory effects of oridonin on angiogenesis in diverse cancer types, including LC (104). Oridonin exhibits potential as an immunostimulatory drug for NK cells, thereby positioning it as a prospective candidate for the treatment of LC. The administration of Oridonin has been observed to improve the cellular cytotoxicity of NK-92MI cells towards tumor cells through the stimulation of degranulation markers and cytotoxic effector molecules. Additionally, it can enhance the upregulation of activation markers on NK-92MI cells, as well as the expression of ligands associated with these indicators in LC cells (87).

#### 3.1.7 Lycopene

Lycopene is a dark-red carotenoid belonging to the tetraterpenoid family and is widely found in a variety of plants, especially ripe red fruits and vegetables such as tomatoes. From an anticancer perspective, people often look to lycopene as a dietary supplement that may help to prevent the occurrence of cancer. Lycopene’s immunomodulatory effects could make it an anticancer agent, as it modulates immune cells to suppress tumor growth and progression (105). Lycopene treatment increases the CD4+/CD8+ ratio in the spleen and promotes IFNγ-expressing CD8+ T cells in tumor tissues. Furthermore, lycopene can assist anti-PD-1 by elevating the IL-1 and IFN-γ levels while also reducing the IL-4 and IL-10 levels in the spleens of mice injected with LLC cells. Upon IFN-γ stimulation, lycopene diminishes PD-L1 expression by activating JAK and repressing AKT phosphorylation (88).

#### 3.1.8 Lupeol

Lupeol is a triterpenoid found in various vegetables and fruits, as well as medicinal plants such as olives, mangos, elms, and *Aloe vera* (L.) Burm. f., *Lupinus micranthus* Guss., etc. Lupeol is an effective inhibitor of proliferating tumour cells and has a powerful anticancer effect against various neoplasms (colorectal, lung, and liver) (106). Plasminogen activator inhibitor-1 (PAI-1) is differentially highly expressed in various types of tumor types, which involved in cancer progression. The lupeol demonstrates inhibitory effects on the synthesis of PAI-1, hence impeding the recruitment of THP-1 macrophages (THP-1 cells that have undergone differentiation into macrophages) towards LC cells. Furthermore, it has been observed that lupeol exhibits the ability to inhibit the polarization of M2 macrophages, resulting in a decrease in the migratory capacity of Lewis LC cells (89).

#### 3.1.9 Paeoniflorin

Paeoniflorin is a monoterpene glycoside isolated from *Paeonia lactiflor*a Pall(also known as *Baishao* in TCM). Paeoniflorin is reported to possess a wide spectrum of anti-tumor effects, including for LC (107). Paeoniflorin can induce tumor cell apoptosis and inhibit tumor proliferation, invasion and metastasis through different mechanisms, and immune regulation be one of the keys to playing these roles.The polarization state and infiltration degree of TAMs in the TME are significantly correlated with cancer treatment and prognosis. Paeoniflorin can affect the cell cycle progression, viability, and migration of lung carcinoma cells by inhibiting the M2 macrophage polarization of TAMs (90).

#### 3.1.10 Bakuchiol

Bakuchiol is a natural meroterpenoid extracted from *Cullen corylifolium* (L.) Medik. Bakuchiol is considered a potential anticancer compound, and its anti-tumor activity against LC has been confirmed *in vitro* (108). Bakuchiol treatment increases the population of cytotoxic immune cells (i.e., M1 macrophages and CD8+ T cells) while also decreasing pro-tumor immune cells (i.e., CD3+ T cells, Tregs, and M2 macrophages). Bakuchiol stimulates the production of anti-inflammatory cytokines, such as IL1-α, IL2, IFN-γ, TNF-α, IL4, and IL10. PD-L1 expression in the tumor is also lowered by Bakuchiol. AKT and STAT3 signaling is inhibited by Bakuchiol (91).

#### 3.1.11 Platycodin D

Platycodin D is a triterpenoid saponin extracted from *Platycodon grandiflorus* (Jacq.) A. DC.(it is also known as *Jiegeng* in TCM). *Jiegeng* is a herb that has been used TCM in China for thousands of years and is commonly found in various tumor treatment TCM formula(*Fufang*). By binding PD-L1 to PD-1 on the surface of T cells, tumor cells can inhibit the immune effect of T cells, leading to immune escape. *Jiegeng* has a role in regulating TIME and can trigger anti-tumor immunity by limiting PD-1 expression in CD8+ T cells (109). Platycodin D decreases the protein level of PD-L1, triggers the extracellular release of PD-L1 in lung carcinoma cells, and enhances IL-2 secretion in T cells (92).

### 3.2 Flavonoids

**Figure 3:**
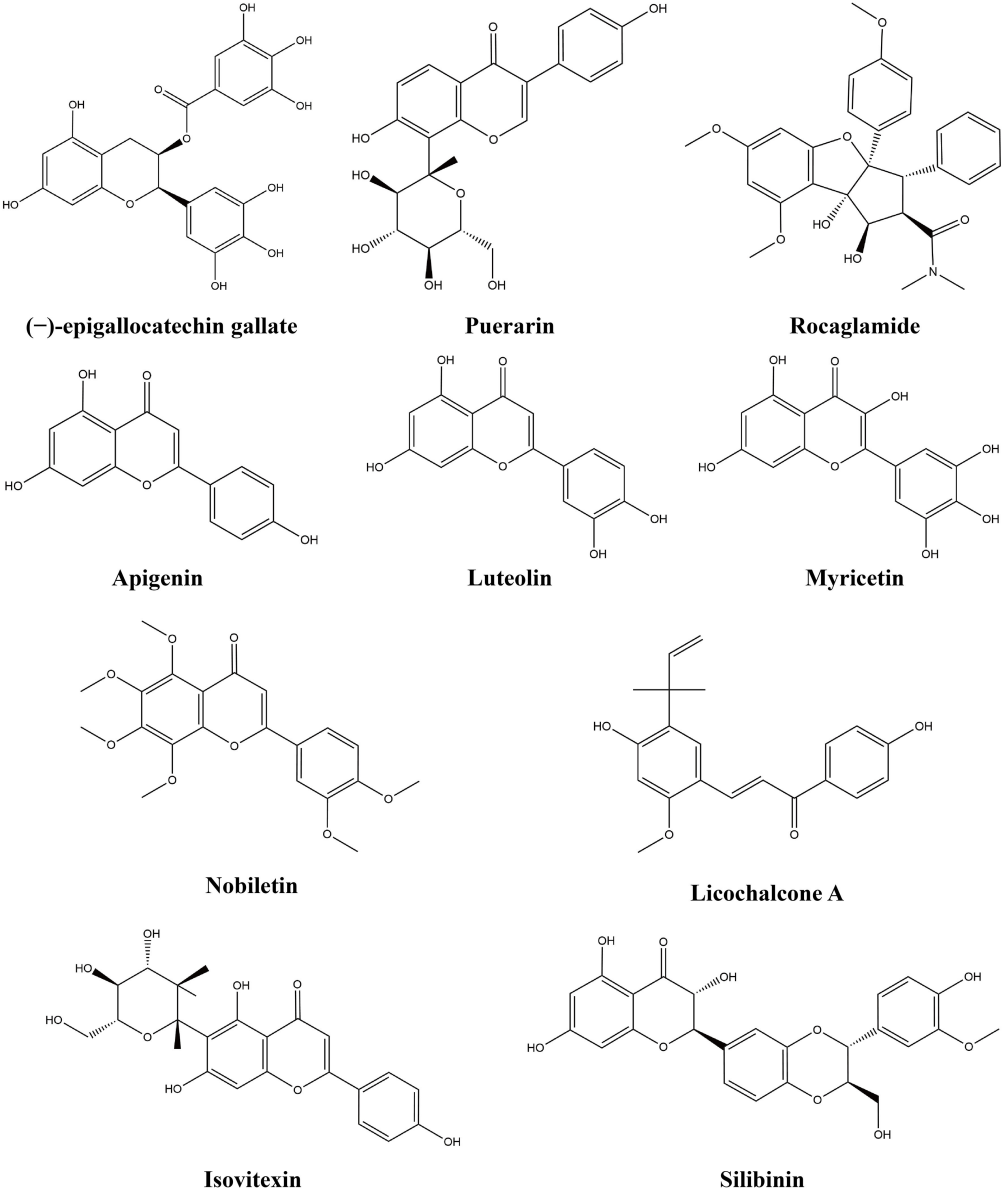
Chemical structures of flavonoids.

Flavonoids are a large class of natural products that have a variety of quantities and complex structures In chemical structure, the basic skeleton of flavonoids is composed of three rings (C6-C3-C6) (110). Many preclinical studies have demonstrated that flavonoids inhibit LC development and can target signaling pathways (111). Flavonoids have diverse and extensive immunomodulatory and anticancer activities (112). This study found that 10 flavonoids exert anti-lung-cancer effects by modulating the TIME. **Table 2** provides the basic information and mechanisms of these 10 flavonoids derived from recent studies on LC, while **Figure 3** presents their chemical structures.

**Table 2:** Basic information and mechanisms of 10 flavonoids from recent studies.

Number	Compounds	Subclass	Molecular Formula	Weight (g/mol)	Main Source	Experimental Model	Dosage (mg/kg/d; μm/L)	Dosing Cycle	Immune Cells/Immune Regulatory Cytokines and Protein	Effects	Reference
1	Rocaglamide	Flavonoid	C29H31NO7	505.6	Aglaia odorata and Aglaia duperreana	LLC mouse model; LLCs and H460, A549, H1975 cells	0, 3, 7 10, and 13 and 1.0; 0, 31.25, 62.5, 125, 250, and 500 nM	21 d; 24 h	NK cells	Enhances NK-cell-mediated killing	(113)
						LLC mouse model; LLCs and A549, H1299, H1975 cells	1 mg/kg/2 d; 0, 25.0, and 50.0	21 d (from day 3); 24 h	NK cells	Enhances infiltration of NK cells	(114)
2	Puerarin	Flavonoid	C21H20O9	416.4	*Pueraria lobata* (Willd.) Ohwi	A549 xenograft mouse lung cancer model	40	30 d	TAMs; IFN-γ, TNF-α and IL-12; IL-10, IL-4 and TGF-β	Increases M1 macrophages, enhances the expression of anti-tumor cytokines (IFN-γ, TNF-α, and IL-12), and reduces the level of pro-tumor cytokines (IL-10, IL-4, and TGF-β).	(115)
3	Nobiletin	Flavonoid	C21H22O8	402.4	Citrus depressa, Citrus reticulata, Citrus sinensis, Citrus limon, etc.	A549, H292, and H460 cells	100, 200	48 h	PD-1	Downregulates PD-L1 expression	(116)
4	(−)-epigallocatechin gallate	Flavonoid	C22H18O11	458.4	Green tea	4-(methylnitrosamino)-1-(3-pyridyl)-1-butanone (NNK)-induced lung cancer mouse model; A549, H1299 and LU-99 cells	Give water containing 0.3% green tea extract (contains 0.85 g/L of catechins in which EGCG is 8%); 10, 50	16 weeks; pretreatment for 3 h	PD-1	Downregulates PD-L1 expression	(117)
5	Myricetin	Flavonoid	C15H10O8	318.23	*Morella rubra* Lour.	A549, H1650 and H460 cells	5, 10, 20	24 h	IFN-γ and PD-L1	Downregulates PD-L1 and IDO1 expression	(118)
6	Licochalcone A	Flavonoid	C21H22O4	338.4	*Glycyrrhiza uralensis* Fisch.	A549 and H1299 cells	5 and 10	6 h	IFN-γ and PD-L1	Abrogates IFN-γ-induced PD-L1 translation	(119)
7	Silibinin	Flavonoid	C25H22O10	482.4	*Silybum marianum* (L.) Gaertn.	H3122 cells	50, 100	24 and 48 h	PD-L1	Downregulates PD-L1 expression	(120)
8	Isovitexin	Flavonoid	C21H20O10	432.4	*Vitex negundo* var. *cannabifolia* (Siebold and Zucc.) Hand.-Mazz.	A549 xenograft mouse lung cancer model; A549 and H1975 cells	20; 25 μg/mL	15 d	CTLs, NK cells; IL-2 and TNF-α	Increases CTLs and NK cell proliferation and activities; promotes the secretion of IL-2 and TNF-α	(121)
9	Luteolin	Flavonoid	C15H10O6	286.24	*Reseda odorata* L.	H358 cell xenograft mouse lung cancer model; H358, H460, H2122, and A549 cells	30; 0, 10, 20, 30, 40, 50	21 d; 4 h	PD-1, PD-L1	Blocks the PD-1/PD-L1 axis, down-regulation PD-L1 expression	(122)
10	Apigenin	Flavonoid	C15H10O5	270.24	*Apium graveolens* L., *Perilla frutescens* (L.) Britton, *Verbena officinalis* L., and *Melissa axillaris* (Benth.) Bakh. f.	H358 cell xenograft mouse lung cancer model; H358, H460, H2122, and A549 cells	30; 0, 10, 20, 30, 40, 50	21 d; 4 h	PD-1, PD-L1	Blocks the PD-1/PD-L1 axis; downregulates PD-L1 expression	(122)

#### 3.2.1 Rocaglamide

Rocaglamide is a flavonoid extract obtained from Aglaia odorata and Aglaia duperreana belonging to the cyclopenta [b]-tetrahydrobenzofurans chemical class. *In vivo* and *in vitro* studies have shown that rocaglamide is a new candidate drug for the treatment of cancer (123). Concerning the structure-specific postulated biogenetic origin of rocamide, it is possible that the cycloaddition of a flavonoid nucleus and a cinnamic acid amide moiety result in the formation of cyclopenta [bc]benzopyran ring system (124).

Rocaglamide can play an anticancer role in NSCLC by promoting NK cell infiltration. There are two main mechanisms by which rocaglamide regulates NK cells, including the autophagy inhibition pathway and the non-autophagy inhibition pathway. UNC-51-like kinase 1 (ULK1), a serine/threonine kinase, plays a key role as an autophagic initiator. In the first pathway, rocaglamide enhances the NK-cell-mediated killing of NSCLC cells by inhibiting autophagy, which is achieved by targeting ULK1 (113). The cyclic GMP-AMP synthase (cGAS) and its downstream signalling effector stimulator of interferon genes (STING) play a crucial role in cancer development. The cGAS/STING signaling pathways has been widely concerned and has become the forefront and hot spot of current research. Rocaglamide activates cGAS/STING signaling pathways in NSCLC cells, leading to the upregulation of CCL5 and CXCL10 and the enhanced infiltration of NK cells (114).

#### 3.2.2 Puerarin

An isoflavone glycoside known as puerarin was extracted from the root of the *Pueraria lobata* (Willd.) Ohwi and is known as *Gegen* in TCM. Puerarin is a naturally occurring medicinal substance that exhibits a range of biological activities, including antioxidative, anti-inflammatory, anti-tumor, immunomodulatory, and neuroprotective properties (125). It is one of the key components of puerarin’s anti-tumor effect, regulating immune cells and cytokines. Puerarin, a negative metastasis regulator of NSCLC, can induce anti-tumor effects by skewing macrophage populations back into the M1 subpopulation. Moreover, puerarin decreases the level of pro-tumor cytokines (IL-10, TGF-β, and IL-4) and increases the expression of anti-tumor cytokines (IFN-γ, IL-12, and TNF-α) (115).

#### 3.2.3 Nobiletin

Nobiletin is a main flavone compound of the peels of citrus fruits, such as *Citrus* depressa, *Citrus* sinensis, *Citrus* reticulata, *Citrus* limon, etc. Numerous pharmacological effects attributed to nobiletin have been researched recently, including NOB’s anti-tumor activity (126). PD-1 is an essential component of the TIME and functions as a receptor that impedes T-cell activation. The EGFR phosphorylation can activates Janus kinase-2 (JAK2) and signal transduction, thereby activating STAT3 to results in the regulation of PD-L1 expression. Nobiletin inhibits the expression of PD-L1 by EGFR/JAK2/STAT3 signaling pathway, thereby enhancing anti-tumor immunity (116).

#### 3.2.4 (−)-Epigallocatechin gallate

The compound (−)-epigallocatechin gallate (EGCG), classified as a flavonoid, is a polyphenolic catechin that constitutes approximately 59% of the overall catechins present in green tea leaves (127). EGCG partially restores T cell activity by inhibiting PD-L1/PD-1 signaling pathway, leading to the inhibition of lung carcinoma cell growth. The mechanism of EGCG-inhibited PD-L1 expression is induced by both IFN-γ and epidermal growth factor (EGF) (117). The role of IFN-γ in tumor immunotherapy is full of paradoxes. IFN-γ can enhance immune function, but it can also accelerate T cell failure by up-regulating PD-L1. The regulation of IFN-γ by EGCG requires further exploration due to its diverse role.

#### 3.2.5 Myricetin

Myricetin is a flavonol compound widely found in many plants. The families Myricaceae, Polygonaceae, Primulaceae, Pinaceae, and Anacardiaceae are the richest sources of myricetin (128). IFN-γ is a major modulator of the TIME and performs a critical function in TIME. Myricetin reverses the effects of IFN-γ-treated LC cells on the survival, CD69 expression, proliferation, and IL-2 production of Jurkat-PD-1 T cells. An essential mechanism underlying the aforementioned therapeutic effects is that IFN-γ induces transcriptional upregulation of PD-L1 and IDO1 via the JAK/STAT/IRF1 axis, which is targeted and inhibited by myricetin (118).

#### 3.2.6 Licochalcone A

Licochalcone A is a flavonoid extracted from *Glycyrrhiza uralensis* Fisch.(also known as *Gancao* in TCM) and presents a wide range of pharmacological effects, including anticancer. The excessive secretion of IFN-γ in the TIME can induce the expression of PD-L1 in tumor cells and promote the immune escape of tumor cells. Licochalcone A abrogates IFN-γ-induced PD-L1 expression via reactive oxygen species (ROS) generation (119).

#### 3.2.7 Silibinin

Silibinin is a natural flavonol isolated from *Silybum marianum* (L.) Gaertn. Silibinin reportedly possesses strong anticancer properties, and immune regulation is one aspect of its anticancer role. NSCLC with anaplastic lymphoma kinase (ALK) gene rearrangement is a specific type of NSCLC. Immune evasion in ALK-positive NSCLC may be facilitated by PD-L1. Silibin reverse acquires resistance and restores sensitivity to crizotinib-resistant tumor cells. Silibinin treatments significantly inhibit the upregulation of the PD-L1 immune checkpoint regulator in crizotinib-refractory LC cells (120).

#### 3.2.8 Isovitexin

Isovitexin is an isomer of vitexin extracted from *Vitex negundo* var. *cannabifolia* (Siebold and Zucc.) Hand.-Mazz. Isovitexin has a variety of biological activities, including anti-cancer. Isovitexin promotes lipopolysaccharide (LPS)- and lectin-stimulated splenocyte proliferation and enhances CTLs and NK cell activities, as well as the secretion of IL-2 and TNF-α (121).

#### 3.2.9 Luteolin (Apigenin)

Luteolin is a flavonoid that was originally isolated from *Reseda odorata* L. It is found in vegetables, medicinal herbs, and fruits. Apigenin is mainly derived from *Apium graveolens* L., but it is also found in other plants such as *Perilla frutescens* (L.) Britton, *Verbena officinalis* L., and *Melissa axillaris* (Benth.) Bakh. f. Luteolin and apigenin can block interactions between PD-1 in T cells and PD-L1 in tumor cells, inhibiting IFN-γ-induced PD-L1 expression (122).

### 3.3 Alkaloids

**Figure 4:**
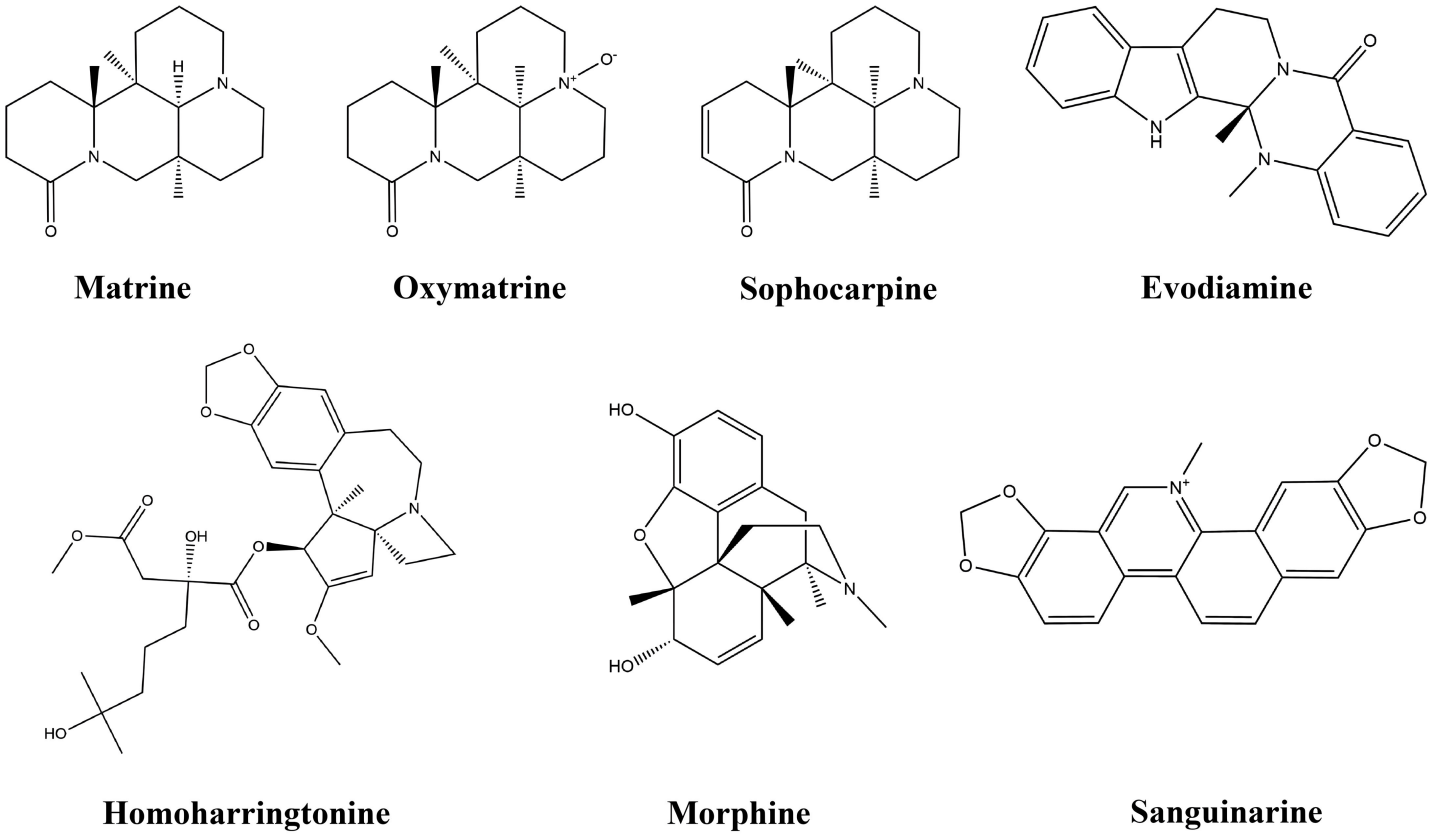
Chemical structures of alkaloids.

Alkaloids are a large group of naturally occurring organic compounds that contain a nitrogen atom or nitrogen atoms in their structures, which cause alkalinity. The acidic tumor microenvironment (ATME) greatly limits the activity of immune cells, which are not conducive to anti-tumor immune responses. Previous studies have shown that low pH in tumors inhibits anticancer immune effectors. In addition, the ATME can contribute to immune evasion (129). Alkaloids can play a therapeutic role by neutralizing the ATME. Alkaloids also serve as a rich resource for drug discovery, and numerous alkaloids screened from medicinal plants have shown antiproliferative and anticancer effects in a wide variety of cancers both *in vitro* and *in vivo* (130). This study found that seven alkaloids exert anti-lung-cancer effects by modulating the TIME. **Table 3** provides the basic information and mechanisms of these seven alkaloids derived from recent studies on LC, while **Figure 4** presents their chemical structures.

**Table 3:** Basic information and mechanisms of 7 alkaloids from recent studies.

Number	Compounds	Subclass	Molecular Formula	Weight (g/mol)	Main Source	Experimental Model	Dosage (mg/kg/d; μm/L)	Dosing Cycle	Immune Cells/Immune Regulatory Cytokines and Protein	Effects	Reference
1	Matrine	Alkaloid	C15H24N2O	248.36	*Sophora flavescens* Aiton	LLCs	0, 1, 2, 4, 8 and 16 μg/mL	24 h	DCs; TNF-α, IL-6, IL-12, and IL-10	Increases the secretion of TNF-α, IL-6, IL-12; decreases the secretion of IL-10; increases the expression of MHC-II, CD80, CD54, CD86; causes autologous mixed T lymphocyte to produce DC-activated killer (DAK) cells	(131)
						LLC mouse model; LLCs, H460 and A549 cells	40 and 80; 20	16 d; 24 h	TAMs	Inhibits the M2 polarization of TAMs	(132)
2	Sanguinarine	Alkaloid	C20H14NO4+	332.3	*Sanguinaria* Canadensis	LLC mouse model; LLCs	5; 0.125, 0.25, 0.5, 1, and 2	20 d, 24 h	Myeloid-derived suppressor cells (MDSCs)	Reduces the accumulation of MDSCs, weakens the immunosuppressive power of MDSCs, promotes the differentiation of MDSCs	(133)
						LLC mouse model	5	3 weeks	TAMs	Modulates M2 macrophage polarization	(134)
3	Homoharringtonine	Alkaloid	C29H39NO9	545.6	Cephalotaxus hainanensis	LL2 mouse model; LL2 and A549 cells	1.25, 2.5; 1, 2, 4, and 8	10 d; 48 h	TIL-B	Promotes the activation of B cells; downregulates the expression of oncogenic proteins (Kras, ERK, Akt, STAT3, CDK4, and CDK6) and tumor suppressors (p21 and RB)	(135)
4	Oxymatrine	Alkaloid	C15H24N2O2	264.36	*Sophora flavescens* Aiton	Male NSCLC patient DCs	1 mg/mL	48 h	DCs, Tregs	Promotes DC maturation; mediates the differentiation of T cells into Tregs	(136)
5	Morphine	Alkaloid	C17H19NO3	285.34	*Papaver somniferum* L.	A549 xenograft mouse lung cancer model; A549 and H460 cells	0.1, 0.3, and 0.5; 1, 10, and 20	3 d; 24 h	PD-L1, TGF-β, IL-10, and IL-2	Upregulates the expression of PD-L1, TGF-β, and IL-10; downregulates the expression of IL-2	(137)
6	Evodiamine	Alkaloid	C19H17N3O	303.4	*Tetradium ruticarpum* (A. Juss.) T. G. Hartley	H1975 mouse lung cancer model, LLC mouse model; H1975 and H1650 cells	10, 20, and 30; 5, 10, and 20	1 week; 48, 72 h	IFN-γ, PD-L1, T cells	Downregulates the expression of PD-L1 and MUC1-C; downregulates the inhibition of IFN-γ-induced PD-L1 expression; downregulates T-cell apoptosis; enhances the effector function of CD8+ T cells; increases the number of CD8+ T cells	(138)
7	Sophocarpine	Alkaloid	C15H22N2O	246.35	*Sophora alopecuroides* Linn	LLC mouse model; LLCs, H1975 and A549 cells	5; 2 mol/mL	18 d; 48 h	PD-L1	Upregulates PD-L1 expression	(139)

#### 3.3.1 Matrine

Matrine is a key tetracycloquinolizindine alkaloid isolated from *Sophora flavescens* Aiton(also known as *Kushen* in TCM). Many studies have been conducted on its anticancer activities against different types of tumour cells, and the results are reasonably acceptable (140).

Toll-like receptors (TLRs) are present and play multiple functions in various immune cell types involved in tumor immunity. TLRS can be expressed on macrophages, neutrophils, DCs, NK cells and mast cells. Matrine exerts an anti-tumor effect by the modulation of the TLR signaling pathway, facilitating the release of immune cytokines, increasing the efficiency of immune cells, and promoting an immunological response. It mainly includes the following aspects: First, matrine interferes with the secretion of immune cytokines, which might decrease the secretion of IL-10 and increase that of IL-6, TNF-α, and IL-12. The second feature is that matrine increases the expression of MHC-II, CD54, CD80, and CD86 on the surface of DCs, which promotes DC differentiation and maturation. In addition, matrine can cause autologous mixed T lymphocytes to produce DC-activated killer (DAK) cells (131). The phosphatidylinositol-3-kinase (PI3K)/protein kinase B (AKT or PKB) signaling pathway serves as a crucial regulator in macrophage survival, migration and proliferation. The anti-cancer and anti-metastasis effects of matrine inhibit EMT induced by M2-like macrophages via the AKT/PI3K signaling pathways, boosting T-cell-mediated anti-tumor immunity by targeting TAMs (132).

#### 3.3.2 Sanguinarine

Sanguinarine, a benzophenanthridine alkaloid obtained from the root of *Sanguinaria canadensis*, exhibits significant potential in the field of cancer treatment due to its potent anticancer properties (141). MDSCs play a key role in inhibiting tumor immune effector cell function, preventing cancer from being attacked by the patient’s immune system. Sanguinarine reduces the MDSC accumulation, weakens the immunosuppressive ability of MDSCs, promotes the differentiation of MDSCs (differentiated into macrophages and DC), and increases the anti-lung-cancer immune response (133). In addition, sanguinarine has the potential to act as a novel regulator of the Wnt/β-catenin signaling pathway, modulating M2 macrophage polarization and inhibiting angiogenesis, both of which have potential application values in LC immunotherapy therapy (134).

#### 3.3.3 Homoharringtonine

Homoharringtonine is a cytotoxic alkaloid that was originally isolated from Cephalotaxus hainanensis. It is a unique agent with a long history of research development. Homotaxine, an approved medication by the Food and Drug Administration (FDA) for the treatment of chronic myeloid leukemia, has been found to exhibit notable inhibitory effects on the expression of NRF2 and ARE-dependent genes in human LC A549 cells. Furthermore, homotaxine has demonstrated its potential as an anti-LC agent, as indicated by numerous studies (142). Homoharringtonine may be a potent immunotherapy drug, against LC associated with Kirsten rats arcomaviral oncogene homolog (KRAS) mutations by directly killing tumour cells and indirectly influencing the TIME. Its influence on the TIME includes two aspects: one promotes the activation of TIL-B, resulting in its anti-tumor activity; the second suppresses the expression of tumor suppressors (RB and p21) and oncogenic proteins (Kras, Akt, ERK, STAT3, CDK6, and CDK4) (135).

#### 3.3.4 Oxymatrine

Oxymatrine, a quinolizidine alkaloid, is a prominent member of the matrine-type alkaloids derived from *Sophora flavescens* Aiton. Numerous investigations have shown that OMT has numerous useful pharmacological qualities, including anticancer effects (143). Oxymatrine may increase the apoptosis of drug-resistant tumor cells by activating DC differentiation and function, thereby enhancing the anti-tumor immune response. Oxymatrine may promote DC maturation and mediate the differentiation of T cells into Tregs (136).

#### 3.3.5 Morphine

Morphine is used as an opioid analgesic to treat acute and chronic moderate-to-severe pain. First discovered in 1806, Serturner isolated the monomer compound morphine from opium poppies, pioneering and promoting a new branch of science that came to be known as alkaloid chemistry (144). Morphine, a common analgesic in clinical practice, is favored for alleviating cancer pain, but it can also control the immune response to tumors. Morphine regulates immune factors (IL-2, IL-10, TGF-β, and PD-L1), thereby promoting tumor immune escape (137).

#### 3.3.6 Evodiamine

Evodiamine is one of the main alkaloid components extracted from *Tetradium ruticarpum* (A. Juss.) T. G. Hartley and has been reported to have anti-tumor activity in human tumour cells (145). MUC1-C is an oncogenic protein that is excessively expressed in cancer. By increasing CD8+ T cells and decreasing the MUC1-C/PD-L1 axis, evodiamine is primarily utilized in the treatment of NSCLC (138).

#### 3.3.7 Sophocarpine

Sophocarpine is one of the representative constituents of quinolizidine alkaloids extracted from *Sophora alopecuroides* Linn. Sophorine is a quinolizidine alkaloid, which has many pharmacological effects, mainly showing strong anti-tumor activity and anti-inflammatory effect. Adenosine A1 receptors (ADORA1) play a role in promoting tumor growth in cancer. Activated transcription factor 3 (ATF3) is abnormally expressed in a variety of cancers and is involved in tumorigenesis. By stimulating the ADORA1/ATF3 axis, sophocarpine increases PD-L1 expression, thereby enhancing the efficacy of PD-L1 inhibitory therapy (139).

### 3.4 Other natural products

**Figure 5:**
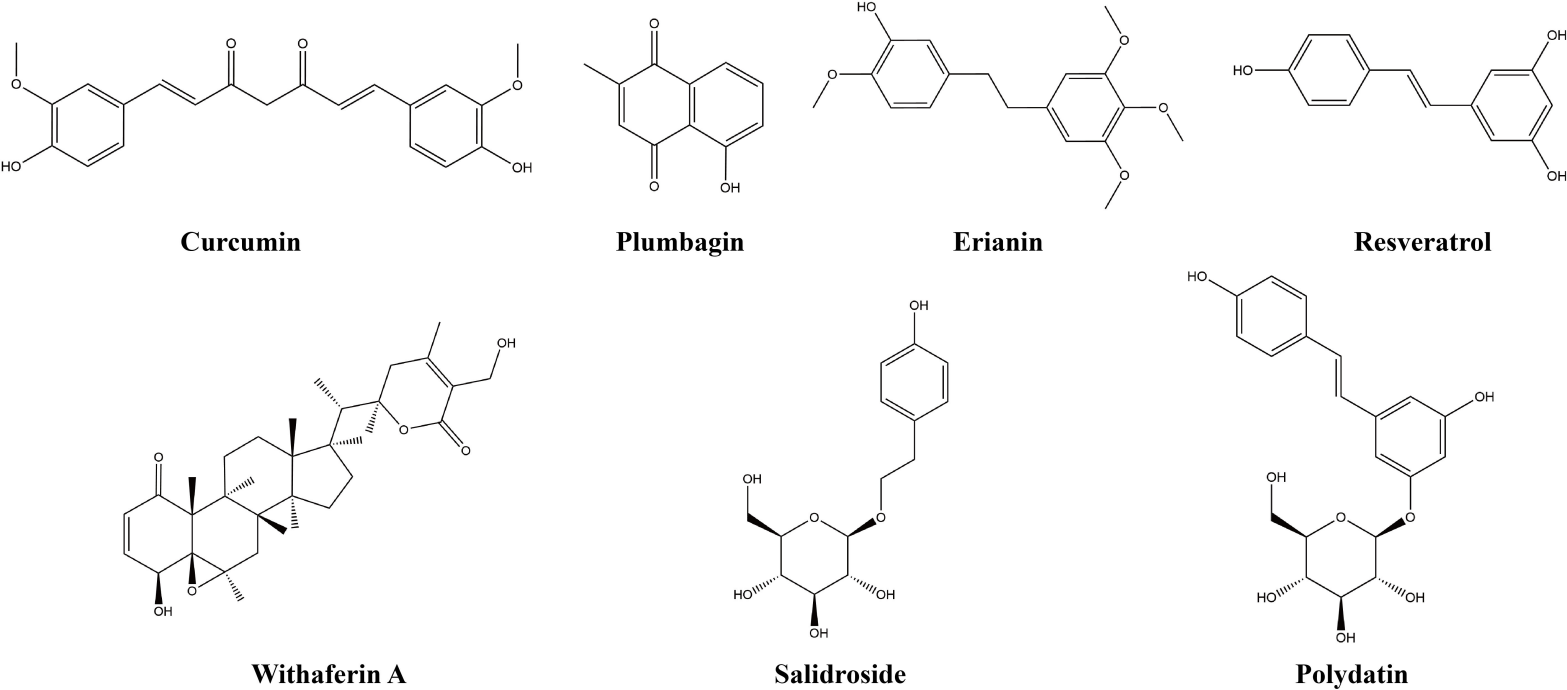
Chemical structures of other natural products.

Other natural products include polyphenols, quinones, steroids, and phenylpropanoids, which are not classified separately given their small quantities, but they are introduced by merging related natural products. This study found that seven other natural products exert anti-lung-cancer effects by modulating the TIME. **Table 4** provides the basic information and mechanisms of these seven additional natural compounds derived from recent studies on LC, while **Figure 5** shows their chemical structures.

**Table 4:** Basic information and mechanisms of 7 additional natural compounds derived from recent studies.

Number	Compounds	Subclass	Molecular Formula	Weight (g/mol)	Main Source	Experimental Model	Dosage (mg/kg/d; μm/L)	Dosing Cycle	Immune Cells/Immune Regulatory Cytokines and Protein	Effects	Reference
1	Curcumin	Polyphenol	C19H24ClNO4	365.8	*Curcuma longa* L.	LLC mouse model; LLCs	50; 20 μM	16 d; 24 h	MDSCs; IL-6	Reduces the accumulation of MDSCs, promotes the maturation and differentiation of MDSCs, inhibits the immunosuppressive function of MDSCs, and decreases the level of IL-6	(146)
						Patients with non-small-cell lung cancer	1.5 g/capsule, twice a day	2 weeks	Tregs	Converts Foxp3+ regulatory T cells into Th 1 cells	(147)
2	Resveratrol	Polyphenol	C14H12O3	228.24	Grapes, mulberries, berries, red wine, peanuts, pines, nuts, etc.	A549 and H1299 cells	20 μM	24 h	TAMs	Inhibits the activation or M2 polarization of TAMs	(148)
						A549 and H1299 cells	20 μM	3 h	PD-L1	Upregulates PD-L1 expression	(149)
3	Polydatin	Quinones	C20H22O8	390.4	*Reynoutria japonica* Houtt.	A549 xenograft mouse lung cancer model	4 mg/d	7 d	Tumor-infiltrating Tumor-infiltrating B (TIL-B)	Decreases TIL-B cell infiltration	(150)
4	Plumbagin	Quinones	C11H8O3	188.18	*Plumbago zeylanica* L.	LLC mouse models	0.5, 1, 2	21 d	CTLs; IFN-γ, TNFα	Increases the number and function of CD8+ T cells; up-regulates the expression of IFN-γ and TNF-α	(151)
5	Withaferin A	Steroid	C28H38O6	470.6	*Withania somnifera* (L.) Dunal (Solanaceae)	LLC mouse model; LLCs, H1650 and A549 cells	4; 0.4 and 0.6 (LLCs and H1650 cells), 2 and 4 (A549 cells)	18 d; 24 h	CTLs; PD-L1	Increases CTL infiltration; upregulates the expression of PD-L1	(152)
6	Salidroside	Phenylpropanoid	C14H20O7	300.3	*Rhodiola rosea* L.	A549 xenograft mouse lung cancer model; A549 cells	40; 10 and 20 μg/mL	1 month; 48 h	CTLs; PD-L1	Suppresses PD-L1; promotes the immune activity of CD8+ T cells	(153)
7	Erianin	Bibenzyl	C18H22O5	318.4	*Dendrobium nobile* Lindl.	LLC mouse model; LLCs, H1975 and A549 cells	10, 35, and 50; 0.0625, 0.125, 0.25, 0.5, and 1 nmol	12 d; 24 h, 48 h	IL-2, TNF-α and IL-10	Upregulates the level of IL-2 and TNF-α; downregulates the level of IL-10	(154)

#### 3.4.1 Curcumin

Curcumin is a lipophilic polyphenol derived from *Curcuma longa* L. that has been documented to have promising anticancer activities, and it is well tolerated in humans (155). MDSCs have a significant role in the TIME as potent immune-suppressive cells. Curcumin has been observed to decrease the accumulation of MDSCs and facilitate the development and differentiation of MDSCs within tumor tissue. Moreover, curcumin also inhibits the immunosuppressive function of MDSCs and decreases the level of IL-6 (146). Curcumin therapy inhibits Tregs and increases Th1 in the peripheral system of LC patients by repressing the gene transcription of Foxp3 and increasing the expression of iIFN-γ (147). Furthermore, curcumin could improve the immune system in LC by inducing efficient T-cell-mediated anti-tumor immunological responses (156).

#### 3.4.2 Resveratrol

Resveratrol, a non-flavonoid polyphenol, was initially identified in the roots of white hellebore (*Veratrum grandiflorum O. Loes*) in 1940, and subsequently isolated from the roots of *Polygonum cuspidatum* in 1963 (157). Resveratrol is mainly found in grapes, mulberries, berries, red wine, peanuts, pines, and nuts. Although resveratrol is a phytochemical known for its antioxidant properties, its anticancer potential has been researched worldwide.

Inhibiting the activation or M2 macrophage polarization of TAMs is an effective cancer therapy. By preventing TAMs from polarizing M2-like, resveratrol suppresses the proliferation of LC (148). Resveratrol inhibits anti-tumor immunity primarily by regulating PD-L1 expression via the Wnt signaling pathway activation pathway (149). In addition, resveratrol can increase TNF-α, IFN-γ, IL-12, and IL-2 expression and increase the cytotoxic effects of CD8+ T-cell expressions in lung squamous cell carcinoma (158).

#### 3.4.3 Polydatin

Polydatin is an anthraquinone component extracted from *Reynoutria japonica* Houtt. and has a variety of biological roles. The therapeutic role of polydatin in LC intervention is positive, and studies have confirmed that it may be a potential therapeutic candidate for treating NSCLC, playing a role by inhibiting proliferation and metastasis (159). Polydatin can improve sensitivity and reduce the adverse effects of radiotherapy through immune regulation. TIL-B cells are found in all phases of LC, implying that B-cells play an important role in the course of the disease. Polydatin significantly decreases radiotherapy-induced tumor B-cell infiltration (150).

#### 3.4.4 Plumbagin

Plumbagin is a plant-derived naphthoquinone mainly obtained from three families, including Plumbaginaceae, Droseraceae, and Ebenaceae (160). The number of CD8+ T cells that infiltrate tumors is shown to rise in mice treated with plumbagin. Furthermore, these cells exhibit promising effector capabilities. Specifically, CD8+ T cells effectively activate phenotypes and increase the effector function of CD8+ T cells by upregulating the production of TNF-α, IFN-γ, and granzyme B (GrzmB). In addition, the proportion of MHC-2/DC cells in mice with tumors treated with plumbagin was increased (151).

#### 3.4.5 Withaferin A

Withaferin A is a pivotal steroidal lactone extracted from *Withania somnifera* (L.) Dunal (Solanaceae), also known as Ashwagandha, was one of the pivotal prehistoric remedies in Ayurveda. Withaferin A is receiving growing attention as a promising anticancer phytochemical, because of its polypharmaceutical medicinal effects, suppressing tumor cell survival, proliferation, motility, metastasis, angiogenesis, and chemosensitization (161).

Withaferin A has been found to trigger ICD in NSCLC cells, leading to an upregulation of PD-L1 expression. Furthermore, withaferin A targets immunosuppressive cells and increases CTL infiltration to raise LC tumor susceptibility to α-PD-L1, which in turn triggers an anti-tumor immune response (152).

#### 3.4.6 Salidroside

Salidroside is a phenylpropanoid glycoside isolated from *Rhodiola rosea* L.(also known as *Hongjingtian* in TCM), which has been used for a long time as adaptogens in TCM (162). Salidroside promotes the immune activity of CD8+ T cells by inhibiting the expression of PD-L1, thereby preventing tumor immune escape in LUAD cells. However, the PD-L1 pathway mediated by circ_0009624 is the key to the above effect (153).

#### 3.4.7 Erianin

Erianin is a natural bibenzyl compound isolated from Dendrobium chrysotoxum Lindl and has been reported to be a prospective natural agent for LC treatment (163). Erianin is abundant in TCM *Shihu, Shihu* is widely used in the treatment of lung diseases. Administering erianin substantially upregulates IL-2 and TNF-α levels, decreases IL-10 levels, and enhances immune function (154).

## 4 Discussion and conclusions

LC is still the leading cause of cancer-related mortality. Surgery, chemotherapy, and radiation therapy are the main conventional treatments commonly used for LC. Cancer immunotherapy has many advantages over chemotherapy and radiation therapy, and immunotherapy has received particular attention because of its favorable efficacy, low-risk ratio, and long-lasting activity (164). The TIME is an important target and pathway for immunotherapy. A variety of immune cells, stromal cells and cytokines within the TME can affect responses to immunotherapy (165). Changes in immune cells, cytokines, and associated receptors in the TIME can affect the development and deterioration of tumors. The anti-tumor effects of natural products are constantly being explored, and they may have value in treating tumors through TIME intervention. A total of 37 natural compounds have been reported to exert immunotherapeutic effects by modulating immune cells, cytokines, or related receptors in the TIME. Many of the natural compounds discussed in this paper can affect the TIME of LC, can improve immune surveillance, have cancer-cell-killing abilities, and have great potential as adjuvant therapies for LC. Tumors can resist chemotherapeutic drugs through various mechanisms, and TIME is crucial to this process. The combining natural compounds and chemotherapeutic drugs has a synergistic effect, which can solve the problem of chemotherapy drug resistance.

Targeting TIME for the treatment of LC has certain regularities, with terpenoids being the most prevalent natural compounds, and 13 species of terpenoids exist. The extensive quantity and diverse array of terpenoids could contribute to this observed phenomenon. In the study of the chemical structure of natural products, we found that there are great differences in the chemical structure of various natural products, and we have not found any obvious similarity and regularity. The sample size currently used is not large enough to support the analysis, which may be one reason for this. Therefore, it is crucial to further augment the sample size in order to comprehensively illuminate the chemical structural attributes of natural products. In the main sources of natural products, some content worth researching and exploring has been found. It is found that many natural products targeting TIME therapy for LC are derived from Chinese Herbal Medicine (CHM). Moreover, these CHM are consistent in TCM classification, that is, most of them belong to the CHM tonic (*Buyi*, Shihu, Hongjingtian, etc.). class, such as *Renshen, Huangqi, Baizhu, Gancao, Baishao, Shihu Hongjingtian, etc.* The CHM exerts its efficacy in cancer treatment by hindering tumor progression and enhancing the immune system of the host organism (166). The tumor elimination mechanism through the restoration of the body’s immune function aligns with the TCM concept of “nourishing positive accumulation and eliminating cancer by itself” (167). These perspectives appear to offer a guiding explanation for the management of LC utilizing TCM.

Tumor-bearing experimental animals are crucial for the preclinical development of cancer drugs. A broad range of tumor models is available, the subcutaneous tumor model is the most frequently utilized model (168). The LLC mouse model and A549 xenograft mouse LC model are the two most common animal models of LC in preclinical studies of natural products against LC. The LLC cell line was first derived from a C57BL mouse that had a tumor in its lung due to the implantation of primary LLC. LLC is highly representative of the human adenocarcinoma subtype of LC and widely used as a model for LC. Human lung adenocarcinoma cells were used to develop the A549 cell line, which is commonly employed to generate xenograft LC models, particularly in NSCLC. Most *in vitro* experiments are also carried out around the above two cell lines, using mature animal or cell models, which also provides more reliable evidence for the effectiveness of using natural products to intervene in LC.

The study on the common/classical signaling pathway for regulating natural products of TIME is a hot topic, but unfortunately, there are no obvious rules to be found. STAT proteins are known as “potential therapeutic targets for altering metabolic rate”. Among them, STAT3-related signaling pathways may have certain developmental value in TIME regulation. During the intervention of triptolide, atractylenolide III,nobiletin and bakuchiol, STAT3-related signaling pathways were all mentioned. The Wnt signaling pathway was mentioned in studies on the regulation of immune cells and immune checkpoints by resveratrol and sanguinarine. But the reference to a generic/classical signaling pathway in the above study seems to be more accidental. Perhaps there is not yet enough content of natural products that regulate TIME-related signaling pathways to provide useful references and enlightening perspectives. Immunotherapies that target PD-1/PD-L1 axis have shown unprecedented success in a wide variety of human cancers (169). Of course, PD-1/PD-L1 axis may be the most concerned common/classical axis in the natural product intervention TIME, and we will introduce it in the next paragraph.

As for the relevant research on using natural products to intervene in the TIME, we found that the target research has concentration characteristics. TAMs are one of the most investigated immune cells about therapies with natural products in TIME. At least ten different natural compounds have been identified to have a role in LC treatment via TAMs. TAMs are crucial components of the TIME and are involved in the progression, metastasis, and proliferation of tumors. The recent advancements in natural compounds in immunomodulation have highlighted their significant potential against cancer (170). In addition, 6 natural products were reported to have anti-cancer effects by regulating CTLs. However, there are fewer studies on natural products targeting other immune cells, so they are not introduced in detail. PD-1 acts as a crucial factor in impeding immune responses and fostering self-tolerance through its regulation of T-cell activity, activation of antigen-specific T-cell apoptosis, and inhibition of Tregs’ apoptosis (171). PD-L1, serving as the primary ligand for PD-1, generates a co-inhibitory signal within activated T cells, hence facilitating T-cell apoptosis, impaired reactivity, and compromised functional capacity (172). The proteins PD-1 and PD-L1 have emerged as significant subjects of study in the field of cancer immune regulation. Researchers have identified at least 17 natural products that exhibit anticancer properties in LC via modulating the activity of these proteins. IFN-γ is a cytokine that is predominantly produced by immune system cells. It plays crucial functions in maintaining tissue equilibrium, as well as in immunological responses and the surveillance of tumors (173). Macrophages are the main physiological targets of IFN-γ, and natural products interfere with the TME by regulating IFN-γ to play an anticancer role. IFN-γ can induce PD-L1 expression on tumor cells, a phenomenon called “adaptive resistance” (174). Many of natural products play an anticancer role by inhibiting IFN-γ secretion/expression and thereby affecting PD-L1. Research has revealed that TNF-α induces diverse oncogenic and tumor-suppressive effects in TME and that dynamic changes in TME could influence the pharmacological action of PD-1/PD-L1 blockers (175). Many natural products have been shown to regulate the secretion of TNF-α, but the subsequent role of TNF-α regulation remains to be further revealed. Attempts to discover a signaling pathway in natural product regulation of TIME have received much attention, but unfortunately have not. The Stat3-related pathway may have some development value, and it has been mentioned in the intervention process of triptolide, atractylenolide III,nobiletin and bakuchiol.

The TIME is a complex system. It is regulated by various signaling pathways, necessitates the interaction of different cell types, and modulates a broad spectrum of cellular responses. Although the numerous studies described in this review have examined the involvement of natural products in cancer mechanisms through TIME interventions, there is still a lack of research on the complex regulatory networks in the TIME formed by multiple pathways. Most natural products work by interfering with one type of immune cell or cytokine, but the incremental effect or crosstalk reaction of different immune cells or cytokines has yet to be revealed. Most studies on TIME intervention using natural products have focused on experiments involving cell lines and subcutaneous tumor mice, so there is a lack of human clinical observation data. The complex structure of natural products can provide effective reference and inspiration for drug development. In addition, the generation of structural analogues to explore structure–activity relationships and optimize natural product leads can be challenging (176). Given a series of factors, including the limited number of studies included in this work and the complexity of the chemical structures of natural products, this study only summarizes the structural commonalities of natural products in the treatment of LC through immune regulation, a subject that needs further exploration. anti-tumor immunotherapy has shown good application prospects in the clinical treatment of LC, but most natural products still need to be taken from their original discovery in basic research to clinical diagnosis and treatment. The use of natural compounds as candidate drugs for new tumor immunotherapies has great development prospects, and it is a path and direction worth exploring in tumor research.

## Author contributions

PY: Writing – original draft. SL: Writing – review & editing. ZL: Writing – original draft. CX: Writing – review & editing.

## Glossary

**Table:**

ATF3	Activated transcription factor 3
ADORA1	adenosine A1 receptors
ALK	anaplastic lymphoma kinase
ADCC	antibody-dependent cell-mediated cytotoxicity
APC	antigen-presenting cell
CAFs	cancer-associated fibroblasts
CCIs	cell-cell interactions
CRT	chaperone protein calreticulin
CHM	Chinese Herbal Medicine
CD	cluster of differentiation
cGAS	cyclic GMP-AMP synthase
CTLs	cytotoxic T-cells
CTLA-4	cytotoxic T-lymphocyte-associated protein 4
DCs	dendritic cells
EGCG	(−)-epigallocatechin gallate
ECM	extracellular matrix
EGFR	epidermal growth factor receptor
EMT	epithelial–mesenchymal transition
GrzmB	granzyme B
HSPs	heat shock proteins
Th cells	helper T cells
JAK2	Janus kinase-2
LUAD	lung adenocarcinoma
LC	lung cancer
LAG3	lymphocyte activation gene 3
ICD	immunogenic cell death
ICB	immune checkpoint blockade
ICI	immune checkpoint inhibitor
IDO	indoleamine 2,3-dioxygenase
ICAM-1	intercellular adhesion molecule-1
IFN-γ	interferon
MHC	major histocompatibility complex
MCs	mast cells
MDSCs	myeloid-derived suppressor cells
NK	natural killer
NSCLC	non-small-cell lung carcinoma
OCT	ocotillol
OA	oleanane
PI3K	phosphatidylinositol-3-kinase
PAI-1	plasminogen activator inhibitor-1
PD-L1	programmed death ligand 1
AKT or PKB	protein kinase B
PPD	protopanaxadiol
PPT	protopanaxatriol
ROS	reactive oxygen species
Tregs	regulatory T cells
RMCRGs	resting MCs
STING	signalling effector stimulator of interferon genes
SCLC	small-cell lung carcinoma
TLSs	tertiary lymphoid structures
TIGIT	T-cell immunoreceptor with immunoglobulin and the ITIM domain
TLRs	toll-like receptors
Trp	tryptophan
TAMs	tumor-associated macrophages
TIME	tumor immune microenvironment
TIL-B	tumor-infiltrating B
TITLs	tumor-infiltrating T lymphocytes
TME	tumor microenvironment
TNF-α	tumor necrosis factor
ULK1	UNC-51-like kinase 1
